# The anxious society’s fertility problem: The promoting effect of physical exercise on mental health—An empirical analysis using CGSS data

**DOI:** 10.1371/journal.pone.0353105

**Published:** 2026-07-23

**Authors:** Jincao Zhai, Dingwu Liu, Mingze Gao, Chunli Han

**Affiliations:** 1 School of Sports Science, Qufu Normal University, Qufu, China; 2 Shandong Sport University, Ji Nan, China; Soochow University, CHINA

## Abstract

The declining willingness to have children is significantly influenced by underlying psychological constraints that cannot be ignored. Providing psychological health support and interventions for the reproductive-age population is crucial for enhancing fertility intentions. Based on data from the 2021 China General Social Survey, this research explores the association between the psychological benefits of physical exercise on individuals’ willingness to bear children. The results indicate a positive correlation between engagement in physical exercise and individual fertility intentions. Specifically, a one-unit higher in level of physical exercise is associated with a 0.099-unit higher level of fertility willingness. Mechanistically, participation in physical exercise is associated with psychological well-being (*β* = 0.0133, *P* < 0.001) and social engagement(*β* = 0.011, *P* < 0.001),these factors are, in turn, associated with higher fertility intentions. Heterogeneity analysis reveals that the positive association between physical exercise and fertility intentions is more pronounced among single individuals with higher educational attainment and income levels *(β = 0.092, β = 0.109, β = 0.095; P < 0.001),*suggesting potential implications for addressing the “fertility-income paradox.”.

## 1. Introduction

Low fertility rates have become a global demographic phenomenon [1].The fertility rates in the United States, Japan, South Korea, Singapore, and certain developed European nations continue to decline, reflecting a widespread issue of low fertility. In recent years, China’s total fertility rate has consistently remained below the internationally recognized threshold of 1.5, significantly below the replacement level, thereby facing the risk of falling into a “low fertility trap [2].” A declining fertility rate triggers issues of population decline and aging, impacting demographic stability and structural balance, th-ereby affecting the sustainable development of future society [3].Addressing the challenge of declining fertility rates and effectively enhancing reproductive levels to promote balanced population development has become an urgent societal issue.

Traditional economic perspectives often attribute declining fertility rates or fertility intentions to high economic costs and the lack of social support systems. However, increasing empirical evidence indicates that purely rational economic models are insufficient to fully explain the complex reproductive decision-making behaviors of individuals. Particularly, they struggle to account for the conflicting sentiments prevalent among women of reproductive age, such as the desire to have children versus the reluctance or fear of childbirth [4].This theoretical shift has prompted scholars to focus on deeper psychological motivations [5].An empirical study on depression and fertility intentions found that individuals exhibiting depressive symptoms often experience emotional distress and negative affect, with diminished perceptions of partner support and family well-being. Consequently, their reproductive intentions are 2.3% lower compared to those without depressive symptoms. This indicates a positive correlation between mental health status and fertility intentions [6].Research in the field of psychology indicates that excessive psychological stress in individuals can trigger intense self-denigration, subsequently diminishing fertility confidence. Providing timely mental health support and emotional assistance to reproductive-age populations can improve their fertility intentions [7].However, existing research predominantly focuses on how to stimulate fertility through external policy interventions such as extending parental leave and increasing childcare subsidies, while paying less attention to how improving individuals’ mental health can foster positive shifts in reproductive attitudes. This bias somewhat diminishes the efficacy of economic levers and constrains the overall effectiveness of fertility promotion policies.

Sports activities are regarded as an efficient and sustainable form of positive psychological intervention,which is associated with mental health.Empirical studies consistently demonstrate that regular physical exercise is associated with higher levels of psychological resilience,life satisfaction, and subjective well-being across multiple dimensions, including physiological regulation, emotional optimization, and behavioral adaptation, while also effectively fostering the development of a positive mindset [8].From this perspective, participatation in physical exercise may may be positively associated with fertility intentions, and may correlate with the establishment of a positive and healthy reproductive mindset.Existing research has examined the relationship between these factors, indicating that participation in sports and leisure activities can fulfill fundamental psychological needs, enhance physical health, and improve psychological well-being, thereby promoting an increase in reproductive intention [9, 10]. Although existing research has addressed the positive psychological correlates of physical exercise with respect to fertility intentions, there remains scope for further in-depth exploration. Existing studies have neither paid sufficient attention to the contextual link between social anxiety, reproductive loss tendencies and subsequent fertility avoidance, nor systematically clarified the antecedent mechanisms and internal pathways linking physical exercise to fertility intentions from a psychological perspective. Relevant theoretical relationships remain to be further explored and clarified.Based on data from the 2021 China General Social Survey (CGSS), this study conducts an in-depth analysis of the complex psychological dilemmas associated with low fertility intentions under an anxiety-inducing social environment.It aims to explore the potential correlation between positive psychological experiences derived from physical exercise and individual fertility intentions.

## 2. Literature review and research hypotheses

### 2.1. The influence of psychological factors on fertility intentions

Psychologist Daniel Kahneman highlights that individual judgment and decision-making are often embedded within complex cognitive schemas [11]. Fertility intention is a psychological representation whereby individuals evaluate their own reproductive,nurturing,and educational capacities.It is not only a product of objective conditions but also the outcome of complex psychological constructs and cognitive reconfigurations, likely influenced by individual psychological traits and tendencies [12].The TDIB reproductive sequence model developed by Miller et al. suggests that individual psychological traits are critical variables for predicting and explaining fertility intentions [13].Fertility anxiety is a specific manifestation of psychological factors influencing reproductive intentions.It refers to the emotional states or experiences,such as worry, tension, and unease that individuals of reproductive age exhibit regarding fertility decisions and related issues [14].Existing studies have fund that such negative emotions not only associated with individuals’ fertility intentions but also potentially reduce reduce them by inducing excessive perception of fertility risks and negative attentional bias, which could in turn affect subjective well-being and life satisfaction [15].

Fertility anxiety is closely linked to the prevailing anxiety traits in contemporary society. Against the backdrop of an era characterized by transition and transformation, the rapid flux of social environments, growing economic downward pressure, intensified career competition, educational involution, and climate and environmental challenges,may jointly contribute to various structural social dilemmas. These factors not only affect people’s daily behavioral choices but also deeply penetrate into individual psychology, tending to foster widespread feelings of anxiety and uncertainty. Various forms of “anxiety syndromes,” such as employment anxiety,education anxiety,and pension anxiety, have emerged accordingly [16]. Fertility anxiety arises within this context and, as its influence expands, has become a latent risk constraining societal reproductive potential and long-term balanced demographic development.Thus, at its root, low fertility intentions can be viewed as a manifestation of generalized social anxiety projected onto the domain of reproduction [17]. Relevant studies on the formation of low fertility rates also indicate that internalized social anxiety, as a subjective psychological barrier, is closely correlated with individuals’ low fertility intentions. For example, River et al. found that women under long-term economic pressure, work-family conflict and future uncertainty are more likely to suffer from anxiety, depression and other negative emotions. Such persistent emotional distress and mental fatigue tend to be correlated with low intrinsic motivation for childbearing and parenting, and may reduce their willingness to have more children [18]. Similarly, Luo Rong et,al. indicated that the extended commuting time resulting from the spatial separation of workplaces and residences in modern society exacerbates psychological stress and tension among residents, causing substantial harm to both physical and mental health. In the context of fertility, this harm manifests as increased concerns about childbearing, delayed childbirth, or fewer children [19].

Under the backdrop of social transformation, chronic anxiety and persistent tension have become prevalent in the mental and psychological states of modern society. The widespread popularity of terms such as “lying flat,” “involution,” “Buddha-like mentality,” and “beast-of-burden workers”serves as vivid manifestations ofthis phenomenon.Such widespread anxious emotions are closely associated with people’s fertility cognition and decision-making, and may lead to certain psychological predicaments.When it comes to childbearing issues, many people are liable to have worries about potential burdens, such as the economic cost of raising children, insufficient family care support and children’s educational pressure.This kind of emotional experience tends to weaken people’s fertility confidence and inner childbearing motivation, and may make people ignore the unique values of parenting such as emotional companionship and spiritual satisfaction.Individuals tend to form inner perceptions such as fear of childbearing and insufficient preparation for parenthood.They are also inclined to agree with views concerning parenting hardships, which may deepen their worries about reproduction and gradually form a self-strengthening psychological tendency.In the social media era, widespread discussions about elite parenting and parenting burnout [20] may further intensify such anxious feelings. These emotional states, combined with actual economic burdens, are closely associated with the downward trend of fertility intentions.

While socioeconomic structural issues represent the core cause of low fertility intentions, the resulting psychological challenges should not be overlooked as a contributing factor. According to the 2023 Blue Book on Mental Health in China, the risk of depression among Chinese adults stands at 10. 16%, while the risk of anxiety disorders reaches 15.8%. Among those who self-rated their mental health as “relatively poor,” the risk of depression was as high as 45. 1%, highlighting the pronounced mental health challenges faced by the population during this period of social transformation [21]. Therefore, it is essential to pay closer attention to the public’s psychological state and mental well-being, and to develop systematic intervention strategies aimed at improving mental health and enhancing psychological welfare. Such efforts are crucial for helping individuals establish a more objective and positive cognitive framework toward parenting.

### 2.2. Research on the relationship between physical exercise and fertility intentions

#### 2.2.1. The association between physical exercise and fertility intentions.

The multifaceted social functions of sports have long been a focus of academic research. Situating the diverse benefits of sports within complex social contexts, relevant studies have revealed that physical exercise is associated with individuals’ subjective perceptions of themselves and the world around them. For instance, empirical evidence indicates that engagement in sports activities is associated with higher life satisfaction, a greater willingness among elderly adults to delay retirement, and lower suicidal ideation among university students [22, 23, 24]. Embodied cognition theory posits that the ways in which the body moves, along with its sensory and motor experiences, shape how we perceive and interpret the world [24]. As an activity fundamentally based on physical movement, sport encompasses diverse bodily practices that may form an embodied foundation for cognitive development, potentially influencing the formation and expansion of schemas related to perception, sensation, thought, and volition [25]. The benefits of physical exercise for psychological and mental well-being may further contribute to holistic human development. Fertility intentions, which essentially reflect the cognitive and attitudinal dispositions of individuals or couples toward childbearing within a given period, are not formed through abstract mental processes alone [26]. Rather, they may be perceived and considered through concrete, practical bodily experiences, rooted in the temporal and spatial dimensions of bodily existence [27].

Based on this reasoning, Hypothesis 1: There is a positive association between physical exercise and individuals’ fertility intentions.

#### 2.2.2. The mediating role of mental health.

Existing research on the relationship between physical exercise and individual cognitive regulation has predominantly focused on its associated benefits in alleviating anxiety, reducing stress, and improving mental health. Representative theoretical frameworks supporting this view include the stress adaptation hypothesis, the HPA axis regulation hypothesis, and the self-efficacy hypothesis. For instance, a study by Guo Kai et al. indicated that physical exercise is associated with fewer negative perceptions of physiological aging among older adults, alongside improved mood disturbances, fewer depressive symptoms, and greater optimism and a proactive mindset, which together relate to a positive self-perception characterized as “defying aging” [28]. As one of the most cost-effective psychological interventions, physical exercise may be related to reduced generalized anxiety affecting fertility-related cognition and decision-making, potentially through lower work and life stress and better mental health, thereby showing a positive association with individual fertility intentions.

At the physiological level, regular physical exercise is associated with optimized stress-regulatory function of the HPA axis, lower excessive amygdala activation, and higher secretion of neurotransmitters such as endorphins and serotonin. These physiological correlates may relate to a higher threshold for anxiety perception, making individuals less prone to excessive panic when confronted with external stressors, which may provide a stable physiological foundation for rational fertility decisions [6]. At the psychological level, engagement in sports is associated with positive psychological traits such as self-esteem, self-confidence, and optimism, as well as enriched emotional experiences including a sense of achievement, happiness, and self-efficacy. These positive emotional states are correlated with broader cognitive scope and greater cognitive flexibility, and they may equip individuals with greater resilience and confidence when confronting uncertainties associated with future parenting [29]. At the social level, the abilities developed through sports practice,such as emotion regulation, stress modulation, and pressure release,may translate into psychological resilience when coping with social pressures. This may relate to a stronger overall sense of control over work and life, enabling individuals to adopt more adaptive cognitive regulation strategies in the face of parenting challenges [30].

Based on this rationale, Hypothesis 2: Physical exercise is positively associated with fertility intentions through its relationship with reduced anxiety, lower stress, and better mental health, suggesting that mental health may play a mediating role in this association.

#### 2.2.3. The Mediating role of social interaction.

Existing studies have confirmed that individual fertility intentions are subject to social influence, which encompasses both behavioral and attitudinal effects from others [31]. For instance, Bernardi’s research demonstrated that social interactions across different spheres,including friends, acquaintances, colleagues, siblings, or neighbors are associated with individuals’ childbearing decisions and behaviors [32]. Social interaction provides individuals with valuable emotional support and parenting-related resources, which may serve as important buffers against fertility-related anxiety, stress, isolation, and uncertainty.

On one hand, when facing stress, effective communication and self-disclosure with relatives and friends may offer emotional support such as understanding, empathy, and comfort, which could be associated with lower psychological anxiety and stronger intrinsic motivation for childbearing [33, 34]. On the other hand, stable social interactions may also help expand individuals’ learning opportunities and resource acquisition channels, thus relating to greater problem-solving capacities, higher confidence, and a stronger sense of control in coping with parenting challenges [33]. As highlighted by Gong Xi et al., frequent interactions among social actors—based on kinship, familial ties, professional relations, and friendships—can generate social capital that provides multidimensional support, including firsthand information on childbearing, emotional comfort, and caregiving assistance, thereby showing associations with both fertility intentions and the timing of childbirth [35].

Physical exercise is widely regarded as being associated with closer interpersonal relationships, higher trust, and greater well-being, and has been described as a “bond of human communication” [36]. In analyzing the social functions of sports, Putnam argued that physical exercise is associated with a broader scope of interpersonal contact, higher social trust, and greater social capital. He further noted that social interactions initiated through sports often occur in a relatively relaxed and informal setting, where individuals tend to be more open and cheerful, making it easier to establish positive connections with others [37]. Huang Qian et al. systematically examined the welfare effects of physical exercise, pointing out that physical exercise is associated with a platform and opportunities for social interaction, broader access to informal resources such as information and support, and a better understanding of others’ choices and trade-offs, thereby relating to more informed decision-making [38].

Based on this reasoning, Hypothesis 3: Physical exercise is positively associated with fertility intentions through its association with greater interpersonal social interactions and more diverse channels for stress relief and resource acquisition.

## 3. Methods

### 3.1. Data

This study utilized data from the 2021 China General Social Survey (CGSS) to examine the relationship between physical exercise and fertility intentions. The initial CGSS sample comprised 8,148 observations. In accordance with the definition of the reproductive-age population (i.e., individuals aged 15–49 years), and given that all CGSS respondents are aged 18 years or older, we restricted the analytical sample to respondents aged 18–49 years by excluding those older than 49 years. We then sequentially excluded observations with missing values on the dependent variable, independent variable, mediating variables, and all control variables. In addition, univariate outliers falling outside the range of mean ± 3 standard deviations were removed. Following these systematic screening procedures, a final valid sample of 3,185 observations was retained for subsequent empirical analysis.

### 3.2. Variable definitions

#### 3.2.1. Dependent variable.

In line with established research practices, this study employs the “ideal number of children” to measure fertility intention [26]. Specifically, the variable is operationalized using the response to survey item A37.1: “If there were no policy restrictions, how many children would you like to have?” The CGSS specially sets the presupposition of “in the absence of policy restrictions,” which to a certain extent eliminates the interference of external policy constraints and helps capture individuals’ genuine fertility preferences.

#### 3.2.2. Independent variable.

The independent variable in this study is physical exercise. This variable is measured using the response to survey item A30.9: “How often have you participated in physical exercise in your free time over the past 12 months?” As the original coding represented a negative indicator (higher values corresponding to lower participation frequency), the responses were reversely coded as 5, 4, 3, 2, and 1 to ensure intuitive interpretation, without altering the fundamental nature or relational structure of the data.This ordinal variable is treated as a continuous variable in the main analysis, which conforms to the standard practice in quantitative social science research. This widely accepted and valid compromise facilitates the adoption of more interpretable parametric statistical models such as linear regression [39].

#### 3.2.3. Mediating variables.

The mediating variables include mental health and social interaction.Mental health was measured using the item: “Over the past four weeks, how often have you felt depressed or downhearted?” Responses were scored such that higher values indicate better mental health status.Social interaction was operationalized based on established measurement approaches in the literature and the structure of the CGSS questionnaire. It was constructed using two items assessing the frequency of social and recreational activities with “neighbors” and with “other friends.” A composite score was generated by averaging the responses to these two items, with higher values reflecting a greater frequency of social interaction.

#### 3.2.4. Control variables.

In addition to the above variables, fertility intentions are also affected by other confounding factors. Therefore, control variables are included in the model to improve estimation accuracy. Referring to factors proven to influence fertility intentions and physical activity in existing studies, control variables are selected from three dimensions: demographic characteristics, economic level and social security status, including gender, ethnicity, religious belief, marital status, educational attainment and social security participation. The specific definition and coding of all variables are shown in Table 1.

**Table 1:** Variable definition and descriptive statistical analysis.

Variable Type	Variable Name	Variable Definition
Dependent Variable	Fertility Intention	Intended number of children (0 or more)
Independent Variable	physical Exercise	Daily = 5, Several times a week = 4, Several times a month = 3, Several times a year or less = 2, Never = 1
Mediating Variables	Mental Health	1 = Always, 2 = Often, 3 = Sometimes, 4 = Rarely, 5 = Never
	Social Interaction	Almost daily = 7, 1–2 times per week = 6, Several times per month = 5, About once per month = 4, Several times per year = 3, Once a year or less = 2, Never = 1
Demographic Characteristics	Gender	Male = 1, Female = 0
	Ethnicity	Ethnic minority = 1, Han Chinese = 0
	Religious Affiliation	No religious affiliation = 1, Has religious affiliation = 0
	Educational Level	Primary school or below = 1, Junior high school = 2, High school or vocational school = 3, College or above = 4
	Marital Status	Married (first marriage with spouse, separated but not divorced, remarried with spouse) = 1, Single (divorced, widowed, cohabitin g, never married) = 0
Household Economic Characteristics	Family Economic Status	Far below and below average = 1, Average = 2, Above and farabove average = 3
Social Attitudes	Social Security	Participates in at least one social security program = 1, No parti cipation in any social security program = 0

## 4. Results

Table 2 presents the descriptive statistics of all variables. The results show that the mean ideal number of children is 1.83, with a median of 2, indicating that the majority of the reproductive-age population prefers to have two children. The mean value for physical exercise is 2.98, with a median of 3, suggesting that most residents engage in physical exercise several times per month. This frequency remains somewhat below the recommended levels outlined in the Physical Activity Guidelines for Chinese Adults (2021), which advise adults to accumulate 2–5 hours of moderate-intensity aerobic activity per week and engage in muscle-strengthening activities on at least two days [40]. Additionally, the mean score for mental health is 4.28, reflecting a generally favorable mental health status among respondents. The mode for social interaction is 6, and the median is 8, indicating a relatively low frequency of interaction with relatives, friends, and neighbors. In the era of intelligent media, social behaviors are increasingly characterized by fatigue and virtualization, with social alienation becoming a more prominent phenomenon [41].

**Table 2:** Descriptive statistical analysis of each variable.

Variable	Mean	Std. Dev.	Mode	Median
Fertility Intention	1.83	1.09	2	2
physical exercise	2.98	1.38	4	3
Mental Health	4.28	4.65	5	4
Social Interaction	7.89	3.01	6	8
Gender	0.43	0.49	0	0
Ethnicity	0.08	0.27	0	0
Religious Affiliation	0.93	0.24	1	1
Educational Level	2.90	1.06	4	3
Marital Status	0.63	0.48	1	1
Family Economic Status	1.74	0.60	2	2
Social Security	0.97	0.17	1	1

Table 3 presents the results of the overall regression analysis.Four regression models were constructed with fertility intention as the dependent variable, physical exercise, mental health, and social interaction as independent variables, while controlling for individual characteristics, family economic status, and social factors. Model 1 serves as the baseline model. Models 2 to 4 were built upon Model 1 by sequentially adding mental health, social interaction, and physical exercise.The regression results in Table 3 indicate that the coefficients for physical exercise are consistently positive (p < 0.001), regardless of whether it is included individually or simultaneously with other variables.This demonstrates a significant positive association between physical exercise and individual fertility intentions, supporting Hypothesis H1.

**Table 3:** Physical activity and fertility intention baseline regression.

Variable	Model 1	Model 2	Model 3	Model 4
physical exercise	0.099^***^ (0.01)	0.085*** (0.009)	0.086*** (0.009)	0.074*** (0.009)
Mental Health		0.218** (0.009)		0.21*** (0.008)
Social Interaction			0.045*** (0.004)	0.038*** (0.004)
Gender	0.04 (0.027)	0.4 （0.025）	0.033 (0.026)	0.017 (0.024)
Ethnicity	0. 173*** (0.05)	0. 144** （0.047）	0. 172*** (0.049)	0. 147** (0.045)
Religious Affiliation	−0. 172** (0.056)	−0. 179*** （0.053）	−0. 175** (0.055)	−0. 167** (0.051)
Educational Level	−0.087** (0.015)	−0.066*** （0.013）	−0.089** (0.015)	−0.083*** (0.013)
Marital Status	0.307*** (0.03)	0.249*** （0.028）	0.305*** (0.029)	0.245*** (0.027)
Family Economic Status	0.018 （0.022）	0.023 （0.021）	0.002 （0.022）	0.002 （0.02）
Social Security	−0. 12 (0.016)	−0.019 (0. 15)	−0.017 (0.015)	−0.021 (0.014)
Constant	1.763 (0.136)	1.259 (0.129)	1.506 (0.136)	0.822 (0.128)
N	3185	3185	3185	3185
_R_^2^	0. 112	0.271	0. 142	0.174

Note: ***P < 0.01, **P < 0.05. The same notation applies throughout.

Table 4 shows the mediating effect test results of mental health and social interaction. The empirical results reveal that physical exercise is significantly positively correlated with fertility intentions (β = 0.756, p < 0.001). Mental health (β = 0.2126, p < 0.001) and social interaction (β = 0.02384, p < 0.001) are also positively associated with fertility intentions. In addition, physical exercise is positively linked to mental health (β = 0.0627, p < 0.05) and social interaction (β = 0.287, p < 0.001). In light of the above regression coefficients, it can be inferred that mental health and social interaction exhibit significant mediating associations between physical exercise and fertility intentions, which supports Hypotheses 2 and 3.

**Table 4:** Regression model analysis of the mediating effect of social interaction and mental health.

Variable	Mental Health	Social Interaction	Fertility Intention
Gender	0.078 (0.51)	0.172 （0.108）	0.017 （0.024）
Ethnicity	0.2 (0.052)	0.067 （0.203）	0.134** （0.044）
Religious Affiliation	0.034 （0.11）	0.069 （0.223）	−0.183** （0.05）
Educational Level	−0.158*** （0.027）	−0.093 （0.056）	−0.078*** （0.012）
Marital Status	0.329*** （0.057）	0.047 （0.121）	0.238*** （0.027）
Family Economic Status	−0.01 （0.043）	0.423*** （0.091）	−0.004 （0.02）
Social Security	0.018 （0.03）	0.132 （0.063）	−0.014 （0.0139）
physical exercise	0.063** （0.018）	0.288*** （0.039）	0.074*** （0.009）
Mental Health			0.213*** （0.008）
Social Interaction			0.038*** （0.004）
Constant	3.467	5.591	0.767
_R_^2^	0.192	0.162	0.294
N	3185	3185	3185

Table 5 reports the mediation effect sizes and confidence intervals for mental health and social interaction.The results indicated a significant total effect of physical exercise on fertility intentions, with an effect size of 0.0984 and a confidence interval excluding zero. The direct effect of physical exercise on fertility intentions was also significant, with an effect size of 0.0741, accounting for 75.3% of the total effect. Furthermore, the total indirect effect was significant (effect size = 0.0244,CI excluding zero).Significant mediating effects were observed through two pathways: the path through mental health (physical exercise→mental health→fertility intentions) with an indirect effect of 0.0133, representing 13.5% of the total effect; and the path through social interaction (physical exercise→social interaction→fertility intentions) with an indirect effect of 0.011, accounting for 11.3% of the total effect.

**Table 5:** Bootstrap analysis of the significance test of the mediating effect and its effect size.

Effect	Path	Effect Size	SE	LLCL	ULCL	Proportion
Total Effect		0.0984	0.0096	0.0796	0.1172	100%
Direct Effect	Direct Path	0.0741	0.0086	0.0571	0.091	75.3%
Total Indirect Effect		0.0244	0.0047	0.0155	0.0338	24.8%
Specific Indirect Effects	Path 1	0.0133	0.0041	0.0055	0.0214	13.5%
	Path 2	0.011	0.002	0.0072	0.0155	11.3%

Table 6 presents the results of the robustness checks.First, the dependent variable was replaced with the intention to have at least one child. Respondents who indicated zero as their ideal number of children were coded as “no fertility intention = 0,” while those reporting one or more children were coded as “having fertility intention = 1.” Accordingly, the regression model was changed to a binary logistic model. The results, presented in Column (1) of Table 6, show that the significance and direction of the coefficients remain largely consistent with the baseline regression, supporting the robustness of the original findings.Second, the independent variable was redefined. Respondents reporting daily, several times per week, or several times per month were recoded as “engaged in sports = 1”, while those reporting several times a year or never were coded as “not engaged in sports = 0”. As shown in Column (2) of Table 6, the correlation between physical exercise and fertility intentions remains statistically consistent, further verifying the reliability of the baseline results.

**Table 6:** Robustness test.

Variable	Replacing Explained Variable (1)	Replacing Explanatory Variable (2)
Fertility Intention	Fertility Intention
physical exercise	0.13*** (0.063)	0.176*** （0.025）
Mental Health	0.051*** （0.003）	0.215*** （0.008）
Social Interaction	0.007** (0.001)	0.039*** (0.004)
Control Variables	Controlled	Controlled
N	3185	3185
R2	0.173	0.286

To further examine the differential effects of physical exercise on fertility intentions across population subgroups, this study conducted grouped regression analyses. As the original dataset contained multiple categories for educational level and family economic status—which are less suitable for grouped regression—these variables were recoded into binary dummy variables. Educational level was categorized as low (secondary education or below =0) and high (college or above =1). Similarly, family economic status was divided into two groups: average or below (=0) and above average (=1). All other variables remained unchanged in specification.

Table 7 presents the results of the heterogeneity analysis.The results reveal significant heterogeneity in the correlation between physical exercise and fertility intentions across family economic status, educational level, and marital status.Specifically, in terms of educational heterogeneity, the correlation magnitude of physical exercise exhibits a clear educational gradient. The coefficient in the high-education group (0.092) is significantly larger than that in the low-education group (0.056), indicating that the positive association between physical exercise and fertility intentions tends to be stronger among people with higher educational attainment.Regarding economic heterogeneity,the regression coefficient increases progressively from 0.069 in the low-income group to 0.109 in the high-income group (p < 0.01), suggesting that this positive correlational trend is more evident among groups with better economic conditions.Such group differences may be associated with varied access to sports resources and different exercise participation frequency across economic groups.Concerning marital status heterogeneity, the correlation strength between physical exercise and fertility intentions varies significantly between married and unmarried individuals.Overall, this linkage appears relatively more evident among unmarried respondents.

**Table 7:** Comparison of differences in education, economics and. marital status between physical exercise.

Variable	（1） Low Education	（2） High Education	（3） Average or Below	（4） Above Average	（5） Married	（6） Unmarried
physical exercise	0.056*** (0.011)	0.092*** (0.016)	0.069*** (0.009)	0. 109*** (0.029)	0.06*** (0.01)	0.095*** (0.017)
Control Variables	Controlled	Controlled	Controlled	Controlled	Controlled	Controlled
N	1895	1290	2855	275	1996	1134
_R_^2^	0.245	0.303	0.29	0.337	0.215	0.305

## 5. Discussion

Research findings indicate a significant positive correlation between physical exercise and individual fertility intentions, a conclusion that aligns with existing study results.Shus as,Zhang Haifeng from the perspective of the intrinsic value of outdoor leisure activities, argue that such activities can promote individuals’ physiological, psychological, and social relationships, as well as their interaction with the environment, which may in turn foster a positive mental state and be associated with residents’ willingness to have additional children [10]. Based on the Second Demographic Transition theory and leisure theory, Deng Weiquan posit that physical exercise fulfills self-oriented needs. When individuals actively engage in sports and achieve need satisfaction, they are more likely to perceive their current environment as suitable for childbearing,which could be related to stronger fertility intentions [9]. Previous studies suggest that sports involvement not only satisfies self-oriented needs but also improves marital and family relationships, creating a psychological and familial environment conducive to childbearing decisions, thereby enhancing individual fertility intentions [42]. Against this backdrop, in situations where external conditions for childbearing are difficult to improve fundamentally in the short term, physical exercise may be seen as a potential psychological empowerment factor that is correlated with “intentional childbearing” and may be associated with internal support for positive fertility decisions.

Although the findings of this study are generally consistent with existing conclusions, the initial linear regression results indicated a weak correlation between the two (r =0.099). Possible reasons for this outcome include:

(1) Observation suggests that most respondents in this sample take physical exercise only several times a month or several times a year, reflecting an overall low level of exercise frequency. At such low participation levels, the psychological benefits brought by physical activity may fail to be fully manifested, which could be one factor associated with the weak linkage with fertility intentions. In line with the dose-response relationship of exercise, it is speculated that only sustained and regular physical exercise might help accumulate psychological benefits to a certain level, which is likely to be linked to a more obvious tendency in fertility willingness [42].
(2) Through an in-depth exploration of the “sports and fertility” nexus, we have identified a potential non-linear relationship between physical exercise and fertility intentions. According to the crowding-out effect theory, engagement in sports requires investments of time and financial resources, which may be indirectly associated with a reduction in the temporal and economic resources available for childbearing and childrearing.This resource shift could be related to relatively lower fertility intentions [43]. But from the perspective of Maslow’s hierarchy of needs, sports participation, as a higher-level need, might serve as an indicator that individuals have already established a certain temporal or material foundation.Simultaneously, considering the functional aspects of sports, regular physical exercise helps alleviate stress, improve emotional states, and enhance self-efficacy, thereby exerting positive influences on individual fertility intentions to some extent. Consequently, the linear model employed in the study may have failed to capture the nonlinear trajectory between physical exercise and fertility intentions, resulting in the observed weak correlation.
(3) Furthermore, random measurement errors inherent in the data collection process, along with the potential influence of other confounding variables, may have collectively contributed to the observed phenomenon.

Physical exercise may be associated with individuals’ fertility intentions, and this association could be partly related to psychological well-being. Social psychology research suggests that the decision to bear children may be partially linked to the possession of greater psychological capital and more resilient psychological traits at both mental and social levels [13]. Individuals with abundant psychological resources possess broader cognitive scope, typically maintaining more positive perceptions of their parenting capabilities while experiencing deeper fulfillment throughout childbearing and childrearing processes. As a component of healthy living, physical exercise is known to be beneficial for maintaining and enhancing physical health. Similarly, its observed associations with anxiety alleviation, stress reduction, and the cultivation of positive psychological attributes may also be relevant to fertility intentions. Through consistent physical exercise, individuals may effectively release occupational and daily pressures and alleviate accumulated psychological burdens, while potentially developing resilient qualities such as self-confidence, optimism, and psychological resilience. These psychological characteristics may correlate with stronger confidence in managing parenting challenges, and could be related to more positive expectations toward future childrearing experiences, which might in turn show associations with fertility intentions.

Physical exercise directly and indirectly enhances individuals’ fertility intentions through the facilitation of social interactions.Existing studies indicate that both offline face-to-face interaction and online virtual social engagement could to some extent correlate with individuals’ childbearing willingness [44]. However, as indicated by earlier analysis, current interactions with relatives and friends mostly occur only several times a year, reflecting clearly insufficient depth and quality of social engagement.In the digital era,public social behavior is characterized by fast-food-style,lightweight,and fragmented interactions [41]. While such shallow social interactions help maintain personal boundaries, they may widen interpersonal gaps, weaken mutual trust, and make it difficult for people to obtain emotional comfort and practical support related to childbearing through daily social contacts. As a spontaneous platform for offline socialization, physical exercise can partly offset the deficiencies of prevailing superficial social modes. When taking part in sports activities together, individuals get chances to interact with diverse groups, perceive others’ perceptions and practical choices regarding childbearing and parenting, and thereby develop diversified understandings of fertility behaviors. Additionally, the relaxed atmosphere of sports activities encourages casual discussions on parenting issues, making it easier for people to gain emotional resonance and practical life experience. Previous research has noted that the pleasant environment of sports activities is conducive to people expanding their social circles and establishing steady interpersonal bonds [45]. Furthermore,the positive interactive experiences and unintentional resource accumulation built through sports activities enhance individuals’ sense of trust and satisfaction with society and their own living environment thereby reducing anxiety and unease caused by environmental uncertainties and infusing more confidence into fertility decisions [28]. The relaxed communicative atmosphere and informal social bonds formed by physical exercise may be linked to individuals’ sense of acceptance and belonging in daily life, which is in turn correlated with lower psychological pressure arising from environmental uncertainty. Such states may further connect with a more rational and calm mindset when making fertility-related decisions, and present a certain positive correlation with increased fertility intentions.

The association between physical exercise and fertility intentions is more pronounced among highly educated, high-income, and unmarried individuals. Conventional studies often suggest a negative relationship between socioeconomic development and fertility intentions—for instance, higher education and income levels typically predict lower fertility intentions, a contradiction known as the “income-fertility paradox” [46]. However,our findings indicate that physical exercise is more strongly associated with fertility intentions specifically within this demographic, suggesting that physical exercise may help mitigate this paradoxical phenomenon.Highly educated, high-income unmarried individuals often face multiple stressors. These stressors are not limited to basic material and security needs but may also extend to societal expectations, work-life balance pressures, and aspirations for self-actualization. The accumulation of such stressors is closely linked to internal conflict and persistent psychological exhaustion among this group. For this demographic, enhancing fertility intentions may depend less on financial subsidies and more on supportive, healthy lifestyle interventions. Physical exercise can be regarded as an effective means of alleviating stress and promoting physical and mental well-being. Through regular exercise, this population may be able to release accumulated work and life pressures, reduce feelings of social isolation, loneliness, and anxiety associated with sedentary, office-centered routines, and gain a sense of achievement, self-efficacy, and happiness. Meanwhile, these positive psychological enhancements may contribute to an improved sense of control over and satisfaction with life, which might, in turn, enable them to approach childbearing from a more rational and forward-looking perspective.

## 6. Recommendations、limitations and future directions

### 6.1. Recommendations

This study indicates that while socioeconomic factors are widely regarded as primary constraints on fertility intentions, a deeper examination of the decision-making logic behind childbearing suggests that psychological barriers, such as anxiety and a diminished sense of meaning, may also represent significant, yet often overlooked, factors associated with low fertility rates. Therefore,enhancing fertility levels may require not only economic and institutional support,but also responsive interventions that address the psychological and emotional needs of reproductive-age individuals in an increasingly uncertain social environment.As an potential psychological intervention,physical exercise may be linked to the systematic renewal of psychological energy and may help individuals accumulate the psychological capital that could be relevant to childbearing decisions.It may therefore be worthwhile to recognize the health benefits derived from sports engagement and to consider integrating them into public health service frameworks. Establishing a well-developed system for promoting sports participation might be associated with positive psychological readiness and intrinsic motivation for childbearing, which could, in turn, relate to long-term balanced population development. Based on the findings, the following recommendations are proposed:

(1)Expand the provision of public fitness services to ensure broader access to the benefits of sports development. This includes continuing to develop sports parks, community sports venues, outdoor fitness trails, and other infrastructure. Emphasis should also be placed on upgrading these spaces with intelligent, diverse, and user-friendly features—such as installing smart fitness equipment and parent-child activity zones—to significantly enhance the accessibility and convenience of physical exercise and enable more people to enjoy the physical and mental health benefits of regular exercise.
(2)Incorporate sports activity as a complementary approach within supportive fertility services. At the community level, full-cycle health promotion courses should be offered, with tailored exercise programs designed for the pre-pregnancy, pregnancy, and postpartum stages. Through scientifically guided physical activity management, these programs can help prevent and alleviate common psychological issues such as prenatal anxiety and postpartum depression, thus enhancing improving psychological resilience throughout the childbearing process.
(3)Develop a national workplace sports strategy to address mental health challenges among employees.The psychological pressures observed among highly educated, high-income, unmarried individuals reflect broader mental health issues within the working population. To this end, a scientifically informed workplace sports development strategy should be established, positioning physical activity as a cornerstone of healthy living for employees. Policy measures may include supporting public and private organizations in outsourcing sports services, establishing employee sports clubs, purchasing fitness equipment, and organizing diverse sports events to reduce barriers to participation. Additionally, providing fitness subsidies or vouchers targeted at employees can lower the financial cost of sports engagement, stimulate interest and enthusiasm for exercise, and help encourage proactive health management and holistic well-being.

### 6.2. Limitations and future directions

Despite the valuable findings, several limitations warrant consideration. The cross-sectional design restricts definitive conclusions regarding causal relationships among variables. Longitudinal studies are required to explore the sustained impact of physical exercise on fertility intentions.The indicator of ideal number of children adopted in this study cannot fully reflect the intensity of fertility intentions. Future studies can adopt multi-dimensional indicators including desired number of children and fertility preference to achieve more accurate effect evaluation. This study does not distinguish the differential effects caused by different participation levels, exercise types and exercise environments. Future studies can conduct more in-depth microscopic analyses to achieve a more comprehensive and objective effect evaluation. In addition, the measurement dimensions of mental health are relatively single, and multi-dimensional measurement tools can be adopted in future research to more comprehensively assess individual mental health status.
