# Supplementary material for: The anxious society’s fertility problem: The promoting effect of physical exercise on mental health—An empirical analysis using CGSS data
Source: PLoS One. 2026 Jul 23;21(7):e0353105. doi: 10.1371/journal.pone.0353105 (PMC13395366; doi:10.1371/journal.pone.0353105)
Supplement: Attachment — Submitted filename: Additional reviewer comments.docx [file pone.0353105.s001.docx]

Dear Authors,

This manuscript addresses a timely and socially relevant topic by examining the association between physical exercise and fertility intentions using a large national dataset (CGSS 2021). The inclusion of mental health and social interaction as potential mediators adds value to the study, and the subgroup analyses provide additional insight into population heterogeneity.

However, several methodological and interpretative issues should be addressed before the manuscript can be considered for publication.

First, the cross-sectional design of the study does not allow for causal inference. Throughout the manuscript, causal language is frequently used (e.g., suggesting that physical exercise “promotes” fertility intentions or improves mental health). The authors should revise the text to consistently reflect associative rather than causal relationships and explicitly acknowledge the possibility of reverse causality.

Second, the measurement of fertility intention based on the “ideal number of children” may not accurately capture actual reproductive intentions. This variable may reflect normative or cultural preferences rather than concrete reproductive plans. This limitation should be more clearly discussed.

Third, the measurement of physical exercise is limited to frequency of participation, without information on intensity, duration, or type. This restricts the interpretation of the findings, as different exercise patterns may have distinct effects. Additionally, the use of self-reported measures introduces potential recall bias.

Fourth, mental health is assessed using a single item related to depressive feelings. This approach does not adequately capture the multidimensional nature of mental health and should be acknowledged as a limitation.

Fifth, some potentially important confounding variables appear to be missing, such as number of existing children, general health status, employment conditions, working hours, and urban/rural residence. The omission of these variables may bias the observed associations and should be explicitly acknowledged.

Sixth, the mediation analysis should be interpreted with caution, as all variables were measured simultaneously. Without temporal ordering, the proposed mediation pathways remain hypothetical and should be framed accordingly.

In addition, some conclusions appear overstated relative to the study design and the magnitude of the observed effects. In particular, broader implications (e.g., demographic or policy-related claims) should be moderated.

Finally, the manuscript would benefit from substantial language editing. Some sections are repetitive, particularly the Introduction and Discussion, and the overall clarity and conciseness could be improved. Professional English editing is recommended.

In summary, the study addresses a relevant topic and has clear potential. However, substantial revisions are required to improve methodological rigor, interpretation, and clarity. I therefore recommend major revision.
