# Supplementary material for: The anxious society’s fertility problem: The promoting effect of physical exercise on mental health—An empirical analysis using CGSS data
Source: PLoS One. 2026 Jul 23;21(7):e0353105. doi: 10.1371/journal.pone.0353105 (PMC13395366; doi:10.1371/journal.pone.0353105)
Supplement: Attachment — Submitted filename: 1Response to Reviewers.docx [file pone.0353105.s003.docx]

Dear Reviewers：

Thank you very much for your thorough review and for pointing out the shortcomings in our work. Your comments have been incredibly helpful and have played a key role in improving the quality of our manuscript. We have carefully considered all of your suggestions and have revised the manuscript accordingly. To facilitate your review, all changes in the revised manuscript have been marked in red. Below are our responses to each of your comments.

Reviewer1：

（1）**The study adopts a cross-sectional design and uses more prudent terms to avoid any causal inference.**

Based on your comments, we recognize that some statements in the original manuscript implied causal inferences, which cannot be validated by a cross-sectional study. Accordingly, we have revised the abstract, introduction, literature review (hypothesis formulation) and discussion sections, and adopted more prudent wording to interpret the research findings objectively and accurately.

Specifically, causal-oriented terms such as *influence, impact, effect* have been replaced with neutral and weakly inferential expressions including *correlation, association, magnitude and correlation strength*. The phrase *be attributed to* is revised to *may be closely related to.*In terms of sentence expression, *"Physical exercise can significantly boost individual fertility willingness"* is revised to *"Physical exercise is positively correlated with individual fertility willingness"*. "*Physical exercise can effectively promote social interaction and improve mental health*" is adjusted to "The study shows that physical exercise is closely linked with positive social interaction and sound mental state".In addition, we have added relevant content to research limitations, stating that causal relationships cannot be confirmed by cross-sectional design, and future studies can adopt longitudinal or experimental designs for further analysis, ensuring the scientificity and rigor of the research. All specific revisions are highlighted in red in the paper for your reference.

**（2）The ideal number of children may not adequately reflect actual fertility intentions.**

Thank you for pointing out this key methodological issue. We fully agree that the "ideal number of children," as a measure of fertility intentions, has certain limitations. We have acknowledged and explicitly discussed this limitation in Section *6.2 "Limitations and future directions,*" noting that "future research could adopt multidimensional measures of fertility intentions to improve construct validity." We would like to explain that this indicator was selected based on data availability and common academic practices. In existing literature, especially studies using data from the Chinese General Social Survey, ideal number of children is one of the most widely adopted indicators to measure fertility intention (doi:10.3390/ijerph18094784).

Besides, robustness tests have been conducted in this study. The results presented in Table 6 verify the consistency of the core correlation that physical exercise is positively associated with ideal number of children, which mitigates potential bias caused by the single measurement indicator. All supplementary contents are marked in red for your review.

**（3）The measurement indicators for physical exercise and mental health were single dimensional and failed to reflect the complexity of the constructs.**

We fully agree with your comment that a single indicator cannot reflect multi-dimensional complex concepts. We have comprehensively discussed and explicitly acknowledged the limitations of the measurement tools for physical exercise and mental health in Section 6.2 Limitations and Future Directions. We also noted that future research should adopt multi-dimensional measurement instruments to more comprehensively assess their correlation.

Similar to the selection of the ideal number of children indicator, the indicators for physical exercise and mental health were chosen based on data availability and prevailing research conventions. Though the current measurements fail to cover all connotations, they fit the research design requirements, ensure data comparability with similar studies, and the analytical results are of valid reference value.

**（4）Explanation of confounding variables**

Thank you for pointing out this important issue. We fully agree that unmeasured confounders such as current number of children, health status, and employment conditions may indeed act as confounding factors influencing the association between physical exercise and the primary outcome variable. In response, we would like to provide the following explanation:In the 2021 Chinese General Social Survey (CGSS) questionnaire, information on current number of children, employment conditions, and urban/rural residence was not collected. Additionally, the measurement scale for working hours was inconsistent with the measurement of our core variables, making it impossible to include these factors in the analysis.The control variables included in this study were selected based on established studies in the literature that have examined factors influencing fertility intentions and physical exercise. These variables were determined across three dimensions—demographic characteristics, economic status, and social security—to cover the main pathways of potential confounding as comprehensively as possible, thereby reducing the risk of bias due to omitted variables.To highlight the adequacy and rationality of the selected control variables, we have revised the discussion in the control variables section to explicitly state that our selection followed multiple mature studies in the field, demonstrating the systematic and standardized nature of the selection process. The specific revisions are as follows:*In addition to the above variables, fertility intentions are also affected by other confounding factors. Therefore, control variables are included in the model to improve estimation accuracy. Referring to factors proven to influence fertility intentions and physical activity in existing studies, control variables are selected from three dimensions: demographic characteristics, economic level and social security status, including gender, ethnicity, religious belief, marital status, educational attainment and social security participation. The specific definition and coding of all variables are shown in Table 1.*

**（5）Cautious interpretation of the mediation analysis**

We fully agree with the valuable suggestion made by the reviewer. All variables in this study were measured cross-sectionally at a single time point, which makes it impossible to establish the temporal order necessary for determining mediation effects. Accordingly, we have revised the manuscript accordingly. We have removed language that implied overly strong causal inferences and have adopted more rigorous wording when interpreting the results of the mediation analysis. The interpretation now focuses primarily on the indirect associations revealed by the study, without drawing definitive causal mediation conclusions, and only objectively describes the associative relationships among the variables.Given the extent of the revisions, it is not feasible to present them all here. All changes have been highlighted in red in the manuscript for your convenience.

Reviewer2：

**（1）Objectively explain that physical exercise is an auxiliary rather than a determining factor**

Thank you for your highly constructive comments. We fully agree with your assessment. This study used physical exercise as an explanatory variable to explore its association with fertility intentions, and we acknowledge that there are indeed limitations in terms of causal identification and logical interpretation. We recognize that physical exercise is essentially an external behavioral manifestation of a healthy lifestyle. Rather than physical exercise itself directly determining fertility choices, it is the underlying healthy lifestyle—characterized by an optimistic outlook and regular daily routines—that serves as the core internal factor driving fertility intentions.In response to your suggestions, we have thoroughly revised the theoretical framework of the manuscript, including the Introduction, Literature Review (hypothesis development), Results interpretation, and Discussion sections. We have toned down the wording that implies a determining role of physical exercise and have redefined it as an auxiliary influencing factor and a psychological buffer. The revised text focuses on elucidating its indirect pathways in shaping fertility intentions—such as alleviating psychological stress, improving physical and mental states, and enhancing personal cognitive attitudes—while objectively describing the associative relationships between variables. We have carefully distinguished between behavioral manifestations and underlying internal mechanisms, thereby avoiding absolute causal inferences.

All specific revisions have been highlighted in red in the manuscript for your ease of review.

**（2）Further refinement of the two pathways**

Thank you for your insightful and constructive comments. We agree with the reviewer that the original description of the pathways was somewhat general and failed to elaborate on the chained mediating psychological transmission process. We have now thoroughly revised the relevant content and refined the internal logic of the two core pathways.

First pathway: Physical exercise may alleviate long‑term work and life stress, regulate negative emotions, reduce fertility‑related loss anxiety, and improve an individual's psychological state, thereby subtly influencing fertility intentions.

Second pathway: Physical exercise creates relaxed offline social interaction opportunities, helping individuals expand social connections, gain more emotional and informational support, alleviate concerns about the uncertainties of childbearing, and thus indirectly influence fertility decisions.

The manuscript now step by step clarifies the progressive psychological mechanisms of "physical exercise → mental health → fertility intentions" and "physical exercise → social interaction → fertility intentions." We have supplemented the logical connections within each step to make the transmission process clear and specific, while maintaining objective wording and avoiding any absolute causal claims.All specific revisions have been highlighted in red in the manuscript for your ease of review.

**（3）Transparency of the sample selection process**

To enhance the methodological rigor and transparency, we have rephrased the data processing procedure. Specifically, we have provided a detailed, step-by-step description of the complete sample selection process and the specific exclusion criteria. This includes clearly specifying the age range restrictions, the rules for excluding cases with missing values on each variable, and the criterion for identifying outliers (values beyond ±3 standard deviations). The complete data processing flow is now fully presented, which effectively improves the transparency of the data processing and ensures the rationality and validity of the final analytical sample. The specific revisions are as follows:*This study employed data from the 2021 China General Social Survey (CGSS), consisting of 8,148 original observations. In line with the definition of the reproductive-age population, namely the age range of 15 to 49 years, and given that all respondents in the CGSS are aged 18 years and above, individuals older than 49 years were excluded, and the sample was restricted to respondents aged 18 to 49 years. Afterwards, observations with missing values in dependent variables, independent variables, mediating variables and all control variables were excluded stepwise. Furthermore, univariate outliers lying outside the range of mean ±3 standard deviations were eliminated. Following the above systematic screening procedures, a final valid sample of 3,185 observations was retained for subsequent empirical analysis.*

**（4）Ordinal variables were treated as continuous variables**

Thank you for pointing out this issue. In this study, physical exercise is an ordinal categorical variable, and it was treated as a continuous variable in the analysis. The rationale for this approach is as follows: the variable's categories are ordered with an increasing gradient, and the differences between adjacent levels are relatively uniform, which aligns with the common academic practice of approximating ordinal variables as continuous. Moreover, previous studies on similar topics have generally adopted this approach, as it facilitates a straightforward exploration of linear associations among variables.Following your suggestion, we have added a justification for this analytical approach along with supporting references in the manuscript. The specific additions are as follows:*This ordinal variable is treated as a continuous variable in the main analysis, which conforms to the standard practice in quantitative social science research. This widely accepted and valid compromise facilitates the adoption of more interpretable parametric statistical models such as linear regression（Frankfort-Nachmias et al., 2020）.*

**（5）Results presentation**

Thank you for pointing out this issue. Following your suggestion, we have standardized the presentation of means and standard deviations for all variables in Table 2, uniformly rounding all values to two decimal places. The table has been reformatted to facilitate easy reading and comparison. In addition, we have thoroughly reviewed the data format of all other tables throughout the manuscript and simultaneously calibrated the corresponding result descriptions to ensure consistent standards of data presentation across the entire paper.

The above are the corresponding revisions made in response to the expert's suggestions. If any of the revisions are inappropriate, or if there are other aspects that require further modification, we sincerely welcome your further comments. It is our great honor to communicate with you. Once again, thank you for your time and valuable suggestions. We look forward to hearing from you.

Sincerely,

The author of PONE-D-25-64152
