# Supplementary material for: The anxious society’s fertility problem: The promoting effect of physical exercise on mental health—An empirical analysis using CGSS data
Source: PLoS One. 2026 Jul 23;21(7):e0353105. doi: 10.1371/journal.pone.0353105 (PMC13395366; doi:10.1371/journal.pone.0353105)
Supplement: Peer review history

# Peer review: Decision Letter 0

**DOI:** 10.1371/journal.pone.0353105.r001

**Authors:** Wanli Zang

11 May 2026

PONE-D-25-64152

The Anxious Society's Fertility ProblemThe Promoting Effect of Physical Exercise on Mental Health—An Empirical Analysis Using CGSS Data

PLOS One

Dear Dr. Han,

Thank you for submitting your manuscript to PLOS ONE. After careful consideration, we feel that it has merit but does not fully meet PLOS ONE’s publication criteria as it currently stands. Therefore, we invite you to submit a revised version of the manuscript that addresses the points raised during the review process.

Please submit your revised manuscript by Jun 25 2026 11:59PM. If you will need more time than this to complete your revisions, please reply to this message or contact the journal office at plosone@plos.org. When you're ready to submit your revision, log on to https://www.editorialmanager.com/pone/ and select the 'Submissions Needing Revision' folder to locate your manuscript file.

Please include the following items when submitting your revised manuscript:

- A letter that responds to each point raised by the academic editor and reviewer(s). You should upload this letter as a separate file labeled 'Response to Reviewers'.
- A marked-up copy of your manuscript that highlights changes made to the original version. You should upload this as a separate file labeled 'Revised Manuscript with Track Changes'.
- An unmarked version of your revised paper without tracked changes. You should upload this as a separate file labeled 'Manuscript'.

If you would like to make changes to your financial disclosure, please include your updated statement in your cover letter. Guidelines for resubmitting your figure files are available below the reviewer comments at the end of this letter.

If applicable, we recommend that you deposit your laboratory protocols in protocols.io to enhance the reproducibility of your results. Protocols.io assigns your protocol its own identifier (DOI) so that it can be cited independently in the future. For instructions see: https://journals.plos.org/plosone/s/submission-guidelines#loc-laboratory-protocols. Additionally, PLOS ONE offers an option for publishing peer-reviewed Lab Protocol articles, which describe protocols hosted on protocols.io. Read more information on sharing protocols at https://plos.org/protocols?utm_medium=editorial-email&utm_source=authorletters&utm_campaign=protocols.

As the corresponding author, your ORCID iD is verified in the submission system and will appear in the published article. PLOS supports the use of ORCID, and we encourage all coauthors to register for an ORCID iD and use it as well. Please encourage your coauthors to verify their ORCID iD within the submission system before final acceptance, as unverified ORCID iDs will not appear in the published article. *Only*  the individual author can complete the verification step; PLOS staff *cannot*  verify ORCID iDs on behalf of authors.

We look forward to receiving your revised manuscript.

Kind regards,

Wanli Zang, Ph.D.

Guest Editor

PLOS One

Journal requirements:

When submitting your revision, we need you to address these additional requirements.

1. Please ensure that your manuscript meets PLOS ONE's style requirements, including those for file naming. The PLOS ONE style templates can be found at

https://journals.plos.org/plosone/s/file?id=wjVg/PLOSOne_formatting_sample_main_body.pdf and

https://journals.plos.org/plosone/s/file?id=ba62/PLOSOne_formatting_sample_title_authors_affiliations.pdf

2. PLOS requires an ORCID iD for the corresponding author in Editorial Manager on papers submitted after December 6th, 2016. Please ensure that you have an ORCID iD and that it is validated in Editorial Manager. To do this, go to ‘Update my Information’ (in the upper left-hand corner of the main menu), and click on the Fetch/Validate link next to the ORCID field. This will take you to the ORCID site and allow you to create a new iD or authenticate a pre-existing iD in Editorial Manager.

If the reviewer comments include a recommendation to cite specific previously published works, please review and evaluate these publications to determine whether they are relevant and should be cited. There is no requirement to cite these works unless the editor has indicated otherwise.

[Note: HTML markup is below. Please do not edit.]

Reviewers' comments:

Reviewer's Responses to Questions

**Comments to the Author**

1. Is the manuscript technically sound, and do the data support the conclusions?

The manuscript must describe a technically sound piece of scientific research with data that supports the conclusions. Experiments must have been conducted rigorously, with appropriate controls, replication, and sample sizes. The conclusions must be drawn appropriately based on the data presented.

Reviewer #1: No

Reviewer #2: Yes

**********

2. Has the statistical analysis been performed appropriately and rigorously?

Reviewer #1: Yes

Reviewer #2: Yes

**********

3. Have the authors made all data underlying the findings in their manuscript fully available?

The PLOS Data policy requires authors to make all data underlying the findings described in their manuscript fully available without restriction, with rare exception (please refer to the Data Availability Statement in the manuscript PDF file). The data should be provided as part of the manuscript or its supporting information, or deposited to a public repository. For example, in addition to summary statistics, the data points behind means, medians and variance measures should be available. If there are restrictions on publicly sharing data—e.g. participant privacy or use of data from a third party—those must be specified.

Reviewer #1: Yes

Reviewer #2: Yes

**********

4. Is the manuscript presented in an intelligible fashion and written in standard English?

PLOS ONE does not copyedit accepted manuscripts, so the language in submitted articles must be clear, correct, and unambiguous. Any typographical or grammatical errors should be corrected at revision, so please note any specific errors here.

Reviewer #1: Yes

Reviewer #2: Yes

**********

5. Review Comments to the Author

Please use the space provided to explain your answers to the questions above. You may also include additional comments for the author, including concerns about dual publication, research ethics, or publication ethics. (Please upload your review as an attachment if it exceeds 20,000 characters)

Reviewer #1: This study addresses a timely and socially relevant question: the potential association between physical exercise and fertility intentions among Chinese adults. The use of a large population-based dataset is an important strength, as is the inclusion of mental health and social interaction as potential explanatory mechanisms.

However, there are several important methodological limitations that should be addressed before the manuscript can be considered for publication.

First, the study uses a cross-sectional design, which prevents any causal inference. In several sections, the authors suggest that physical exercise increases fertility intentions, improves mental health, or reduces fertility-related anxiety. However, with cross-sectional data, only associations can be identified. Therefore, the authors should replace causal language with more cautious terms such as “is associated with” or “may be associated with.”

Second, the dependent variable used (“ideal number of children”) may not adequately represent actual fertility intentions. This measure may reflect normative or cultural preferences rather than concrete reproductive plans.

Third, the measurement of physical exercise is limited to participation frequency, without information on intensity, duration, modality, or weekly volume. Similarly, mental health was assessed using only a single question related to feelings of sadness or depression, which may not adequately capture the complexity of this construct.

In addition, several potentially important confounding variables appear to be missing from the models, including current number of children, health status, employment conditions, urban versus rural residence, and working hours. The omission of these variables may introduce residual confounding.

The mediation analyses should also be interpreted cautiously because all variables were measured at the same time. Therefore, the temporal order necessary to support mediation cannot be established.

Finally, the manuscript contains problems related to language, repetition, and English fluency. Professional language editing is strongly recommended to improve clarity, conciseness, and coherence.

Overall, the study has merit and potential, but it requires substantial revision to better align the interpretation of the findings with the limitations of the design and data.

Reviewer #2: Dear Authors,Thank you for the opportunity to review your manuscript titled: *The Anxious Society's Fertility Problem: The Promoting Effect of Physical Exercise on Mental Health—An Empirical Analysis Using CGSS Data*. This study attempts to introduce “physical exercise participation” as a health behavior into the explanatory framework of the psychological mechanisms underlying low fertility intentions, thereby addressing the limitations of previous studies that have focused solely on economic and policy perspectives. The topic has significant practical relevance and demonstrates theoretical innovation. The data are reliable and the methodology is generally sound. However, in its current version, the manuscript has several issues related to theoretical development, data transparency, presentation of results, and language expression.Theoretical Development and Mechanism Elaboration1. This study uses physical exercise participation as an explanatory variable to explore its effect on fertility intentions, but there are issues concerning logic and causal identification. Physical exercise participation represents a healthy lifestyle. It may be that a healthy lifestyle (characterized by positivity, optimism, and regularity) enhances fertility intentions, rather than physical exercise participation directly promoting fertility intentions. It is recommended that the authors more objectively explain the role of physical exercise participation in the theoretical development section, positioning it as a “supportive” or “psychological buffering” factor.2. The articulation of how the proposed mechanisms—mental health and social interaction—specifically alleviate “fertility loss anxiety” remains relatively abstract. The specific psychological pathways from “physical exercise participation → mental health → fertility intentions” and “physical exercise participation → social interaction → fertility intentions” need further refinement.Data Transparency1. The authors report substantial sample loss (from 8,148 to 3,085), but state only that cases with missing data were deleted, without providing specific deletion criteria. This lack of transparency regarding data processing undermines the validity of the sample.2. The variable for physical exercise participation is ordinal but is treated as continuous in the analysis without justification. The authors should provide supplementary explanation for this treatment.Presentation of Results1. Please check the standard deviations and means of the variables in Table 2 and keep a consistent number of decimal places (e.g., two) to facilitate readability.

2

**********

6. PLOS authors have the option to publish the peer review history of their article (what does this mean?). If published, this will include your full peer review and any attached files.

If you choose “no”, your identity will remain anonymous but your review may still be made public.

**Do you want your identity to be public for this peer review?**  For information about this choice, including consent withdrawal, please see our Privacy Policy.

Reviewer #1: No

Reviewer #2: **Yes:** zhouxiao

**********

[NOTE: If reviewer comments were submitted as an attachment file, they will be attached to this email and accessible via the submission site. Please log into your account, locate the manuscript record, and check for the action link "View Attachments". If this link does not appear, there are no attachment files.]

To ensure your figures meet our technical requirements, please review our figure guidelines: https://journals.plos.org/plosone/s/figures

You may also use PLOS’s free figure tool, NAAS, to help you prepare publication quality figures: https://journals.plos.org/plosone/s/figures#loc-tools-for-figure-preparation.

NAAS will assess whether your figures meet our technical requirements by comparing each figure against our figure specifications.

Attachment Submitted filename: Additional reviewer comments.docx

# Author comment: Author response to Decision Letter 1

**DOI:** 10.1371/journal.pone.0353105.r002

25 May 2026

Dear Reviewers

Thank you very much for your thorough review and for pointing out the shortcomings in our work. Your comments have been incredibly helpful and have played a key role in improving the quality of our manuscript. We have carefully considered all of your suggestions and have revised the manuscript accordingly. To facilitate your review, all changes in the revised manuscript have been marked in red. Below are our responses to each of your comments.

Reviewer1

�1�The study adopts a cross-sectional design and uses more prudent terms to avoid any causal inference.

Based on your comments, we recognize that some statements in the original manuscript implied causal inferences, which cannot be validated by a cross-sectional study. Accordingly, we have revised the abstract, introduction, literature review (hypothesis formulation) and discussion sections, and adopted more prudent wording to interpret the research findings objectively and accurately.

Specifically, causal-oriented terms such as influence, impact, effect have been replaced with neutral and weakly inferential expressions including correlation, association, magnitude and correlation strength. The phrase be attributed to is revised to may be closely related to.In terms of sentence expression, "Physical exercise can significantly boost individual fertility willingness" is revised to "Physical exercise is positively correlated with individual fertility willingness". "Physical exercise can effectively promote social interaction and improve mental health" is adjusted to "The study shows that physical exercise is closely linked with positive social interaction and sound mental state".In addition, we have added relevant content to research limitations, stating that causal relationships cannot be confirmed by cross-sectional design, and future studies can adopt longitudinal or experimental designs for further analysis, ensuring the scientificity and rigor of the research. All specific revisions are highlighted in red in the paper for your reference.

�2�The ideal number of children may not adequately reflect actual fertility intentions.

Thank you for pointing out this key methodological issue. We fully agree that the "ideal number of children," as a measure of fertility intentions, has certain limitations. We have acknowledged and explicitly discussed this limitation in Section 6.2 "Limitations and future directions," noting that "future research could adopt multidimensional measures of fertility intentions to improve construct validity." We would like to explain that this indicator was selected based on data availability and common academic practices. In existing literature, especially studies using data from the Chinese General Social Survey, ideal number of children is one of the most widely adopted indicators to measure fertility intention (doi:10.3390/ijerph18094784).

Besides, robustness tests have been conducted in this study. The results presented in Table 6 verify the consistency of the core correlation that physical exercise is positively associated with ideal number of children, which mitigates potential bias caused by the single measurement indicator. All supplementary contents are marked in red for your review.

�3�The measurement indicators for physical exercise and mental health were single dimensional and failed to reflect the complexity of the constructs.

We fully agree with your comment that a single indicator cannot reflect multi-dimensional complex concepts. We have comprehensively discussed and explicitly acknowledged the limitations of the measurement tools for physical exercise and mental health in Section 6.2 Limitations and Future Directions. We also noted that future research should adopt multi-dimensional measurement instruments to more comprehensively assess their correlation.

Similar to the selection of the ideal number of children indicator, the indicators for physical exercise and mental health were chosen based on data availability and prevailing research conventions. Though the current measurements fail to cover all connotations, they fit the research design requirements, ensure data comparability with similar studies, and the analytical results are of valid reference value.

�4�Explanation of confounding variables

Thank you for pointing out this important issue. We fully agree that unmeasured confounders such as current number of children, health status, and employment conditions may indeed act as confounding factors influencing the association between physical exercise and the primary outcome variable. In response, we would like to provide the following explanation:In the 2021 Chinese General Social Survey (CGSS) questionnaire, information on current number of children, employment conditions, and urban/rural residence was not collected. Additionally, the measurement scale for working hours was inconsistent with the measurement of our core variables, making it impossible to include these factors in the analysis.The control variables included in this study were selected based on established studies in the literature that have examined factors influencing fertility intentions and physical exercise. These variables were determined across three dimensions—demographic characteristics, economic status, and social security—to cover the main pathways of potential confounding as comprehensively as possible, thereby reducing the risk of bias due to omitted variables.To highlight the adequacy and rationality of the selected control variables, we have revised the discussion in the control variables section to explicitly state that our selection followed multiple mature studies in the field, demonstrating the systematic and standardized nature of the selection process. The specific revisions are as follows:In addition to the above variables, fertility intentions are also affected by other confounding factors. Therefore, control variables are included in the model to improve estimation accuracy. Referring to factors proven to influence fertility intentions and physical activity in existing studies, control variables are selected from three dimensions: demographic characteristics, economic level and social security status, including gender, ethnicity, religious belief, marital status, educational attainment and social security participation. The specific definition and coding of all variables are shown in Table 1.

�5�Cautious interpretation of the mediation analysis

We fully agree with the valuable suggestion made by the reviewer. All variables in this study were measured cross-sectionally at a single time point, which makes it impossible to establish the temporal order necessary for determining mediation effects. Accordingly, we have revised the manuscript accordingly. We have removed language that implied overly strong causal inferences and have adopted more rigorous wording when interpreting the results of the mediation analysis. The interpretation now focuses primarily on the indirect associations revealed by the study, without drawing definitive causal mediation conclusions, and only objectively describes the associative relationships among the variables.Given the extent of the revisions, it is not feasible to present them all here. All changes have been highlighted in red in the manuscript for your convenience.

Reviewer2

�1�Objectively explain that physical exercise is an auxiliary rather than a determining factor

Thank you for your highly constructive comments. We fully agree with your assessment. This study used physical exercise as an explanatory variable to explore its association with fertility intentions, and we acknowledge that there are indeed limitations in terms of causal identification and logical interpretation. We recognize that physical exercise is essentially an external behavioral manifestation of a healthy lifestyle. Rather than physical exercise itself directly determining fertility choices, it is the underlying healthy lifestyle—characterized by an optimistic outlook and regular daily routines—that serves as the core internal factor driving fertility intentions.In response to your suggestions, we have thoroughly revised the theoretical framework of the manuscript, including the Introduction, Literature Review (hypothesis development), Results interpretation, and Discussion sections. We have toned down the wording that implies a determining role of physical exercise and have redefined it as an auxiliary influencing factor and a psychological buffer. The revised text focuses on elucidating its indirect pathways in shaping fertility intentions—such as alleviating psychological stress, improving physical and mental states, and enhancing personal cognitive attitudes—while objectively describing the associative relationships between variables. We have carefully distinguished between behavioral manifestations and underlying internal mechanisms, thereby avoiding absolute causal inferences.

All specific revisions have been highlighted in red in the manuscript for your ease of review.

�2�Further refinement of the two pathways

Thank you for your insightful and constructive comments. We agree with the reviewer that the original description of the pathways was somewhat general and failed to elaborate on the chained mediating psychological transmission process. We have now thoroughly revised the relevant content and refined the internal logic of the two core pathways.

First pathway: Physical exercise may alleviate long‑term work and life stress, regulate negative emotions, reduce fertility‑related loss anxiety, and improve an individual's psychological state, thereby subtly influencing fertility intentions.

Second pathway: Physical exercise creates relaxed offline social interaction opportunities, helping individuals expand social connections, gain more emotional and informational support, alleviate concerns about the uncertainties of childbearing, and thus indirectly influence fertility decisions.

The manuscript now step by step clarifies the progressive psychological mechanisms of "physical exercise → mental health → fertility intentions" and "physical exercise → social interaction → fertility intentions." We have supplemented the logical connections within each step to make the transmission process clear and specific, while maintaining objective wording and avoiding any absolute causal claims.All specific revisions have been highlighted in red in the manuscript for your ease of review.

�3�Transparency of the sample selection process

To enhance the methodological rigor and transparency, we have rephrased the data processing procedure. Specifically, we have provided a detailed, step-by-step description of the complete sample selection process and the specific exclusion criteria. This includes clearly specifying the age range restrictions, the rules for excluding cases with missing values on each variable, and the criterion for identifying outliers (values beyond ±3 standard deviations). The complete data processing flow is now fully presented, which effectively improves the transparency of the data processing and ensures the rationality and validity of the final analytical sample. The specific revisions are as follows:This study employed data from the 2021 China General Social Survey (CGSS), consisting of 8,148 original observations. In line with the definition of the reproductive-age population, namely the age range of 15 to 49 years, and given that all respondents in the CGSS are aged 18 years and above, individuals older than 49 years were excluded, and the sample was restricted to respondents aged 18 to 49 years. Afterwards, observations with missing values in dependent variables, independent variables, mediating variables and all control variables were excluded stepwise. Furthermore, univariate outliers lying outside the range of mean ±3 standard deviations were eliminated. Following the above systematic screening procedures, a final valid sample of 3,185 observations was retained for subsequent empirical analysis.

�4�Ordinal variables were treated as continuous variables

Thank you for pointing out this issue. In this study, physical exercise is an ordinal categorical variable, and it was treated as a continuous variable in the analysis. The rationale for this approach is as follows: the variable's categories are ordered with an increasing gradient, and the differences between adjacent levels are relatively uniform, which aligns with the common academic practice of approximating ordinal variables as continuous. Moreover, previous studies on similar topics have generally adopted this approach, as it facilitates a straightforward exploration of linear associations among variables.Following your suggestion, we have added a justification for this analytical approach along with supporting references in the manuscript. The specific additions are as follows:This ordinal variable is treated as a continuous variable in the main analysis, which conforms to the standard practice in quantitative social science research. This widely accepted and valid compromise facilitates the adoption of more interpretable parametric statistical models such as linear regression�Frankfort-Nachmias et al., 2020�.

�5�Results presentation

Thank you for pointing out this issue. Following your suggestion, we have standardized the presentation of means and standard deviations for all variables in Table 2, uniformly rounding all values to two decimal places. The table has been reformatted to facilitate easy reading and comparison. In addition, we have thoroughly reviewed the data format of all other tables throughout the manuscript and simultaneously calibrated the corresponding result descriptions to ensure consistent standards of data presentation across the entire paper.

The above are the corresponding revisions made in response to the expert's suggestions. If any of the revisions are inappropriate, or if there are other aspects that require further modification, we sincerely welcome your further comments. It is our great honor to communicate with you. Once again, thank you for your time and valuable suggestions. We look forward to hearing from you.

Sincerely,

The author of PONE-D-25-64152

Attachment Submitted filename: 1Response to Reviewers.docx

# Peer review: Decision Letter 1

**DOI:** 10.1371/journal.pone.0353105.r003

**Authors:** Wanli Zang

19 Jun 2026

The Anxious Society's Fertility ProblemThe Promoting Effect of Physical Exercise on Mental Health—An Empirical Analysis Using CGSS Data

PONE-D-25-64152R1

Dear Dr. Han,

We’re pleased to inform you that your manuscript has been judged scientifically suitable for publication and will be formally accepted for publication once it meets all outstanding technical requirements.

Within one week, you’ll receive an e-mail detailing the required amendments. When these have been addressed, you’ll receive a formal acceptance letter and your manuscript will be scheduled for publication.

An invoice will be generated when your article is formally accepted. Please note, if your institution has a publishing partnership with PLOS and your article meets the relevant criteria, all or part of your publication costs will be covered. Please make sure your user information is up-to-date by logging into Editorial Manager at Editorial Manager® and clicking the ‘Update My Information' link at the top of the page. For questions related to billing, please contact billing support.

If your institution or institutions have a press office, please notify them about your upcoming paper to help maximize its impact. If they’ll be preparing press materials, please inform our press team as soon as possible -- no later than 48 hours after receiving the formal acceptance. Your manuscript will remain under strict press embargo until 2 pm Eastern Time on the date of publication. For more information, please contact onepress@plos.org.

Kind regards,

Wanli Zang, Ph.D.

Guest Editor

PLOS One

Additional Editor Comments (optional):

Reviewers' comments:

Reviewer's Responses to Questions

**Comments to the Author**

1. If the authors have adequately addressed your comments raised in a previous round of review and you feel that this manuscript is now acceptable for publication, you may indicate that here to bypass the “Comments to the Author” section, enter your conflict of interest statement in the “Confidential to Editor” section, and submit your "Accept" recommendation.

Reviewer #1: All comments have been addressed

Reviewer #2: All comments have been addressed

**********

2. Is the manuscript technically sound, and do the data support the conclusions?

The manuscript must describe a technically sound piece of scientific research with data that supports the conclusions. Experiments must have been conducted rigorously, with appropriate controls, replication, and sample sizes. The conclusions must be drawn appropriately based on the data presented.

Reviewer #1: Yes

Reviewer #2: Yes

**********

3. Has the statistical analysis been performed appropriately and rigorously?

Reviewer #1: Yes

Reviewer #2: Yes

**********

4. Have the authors made all data underlying the findings in their manuscript fully available?

The PLOS Data policy requires authors to make all data underlying the findings described in their manuscript fully available without restriction, with rare exception (please refer to the Data Availability Statement in the manuscript PDF file). The data should be provided as part of the manuscript or its supporting information, or deposited to a public repository. For example, in addition to summary statistics, the data points behind means, medians and variance measures should be available. If there are restrictions on publicly sharing data—e.g. participant privacy or use of data from a third party—those must be specified.

Reviewer #1: Yes

Reviewer #2: Yes

**********

5. Is the manuscript presented in an intelligible fashion and written in standard English?

PLOS ONE does not copyedit accepted manuscripts, so the language in submitted articles must be clear, correct, and unambiguous. Any typographical or grammatical errors should be corrected at revision, so please note any specific errors here.

Reviewer #1: Yes

Reviewer #2: Yes

**********

6. Review Comments to the Author

Please use the space provided to explain your answers to the questions above. You may also include additional comments for the author, including concerns about dual publication, research ethics, or publication ethics. (Please upload your review as an attachment if it exceeds 20,000 characters)

Reviewer #1: Thank you to the authors for the careful revision of the manuscript.

The authors have adequately addressed the major concerns raised during the previous round of review. In particular, the interpretation of the findings has been substantially improved through the replacement of causal language with associative language, which is more appropriate for a cross-sectional study design.

The methodological procedures are now described more clearly, including sample selection criteria, variable definitions, robustness analyses, and study limitations. The authors have also explicitly acknowledged the limitations inherent to the cross-sectional design and have appropriately discussed the implications of their findings.

The statistical analyses are appropriate for the stated objectives, and the conclusions are generally supported by the data presented. Ethical considerations and data availability have also been adequately addressed.

Although some minor language and stylistic issues remain, they do not affect the overall clarity of the manuscript or the validity of the scientific findings.

Overall, I believe that the manuscript meets the standards of methodological rigor, transparency, and scientific soundness required by PLOS ONE and is suitable for publication.

Reviewer #2: (No Response)

**********

7. PLOS authors have the option to publish the peer review history of their article (what does this mean?). If published, this will include your full peer review and any attached files.

If you choose “no”, your identity will remain anonymous but your review may still be made public.

**Do you want your identity to be public for this peer review?**  For information about this choice, including consent withdrawal, please see our Privacy Policy.

Reviewer #1: **Yes:** Carla Christina Ade Caldas

Reviewer #2: No

**********

# Editor report: Acceptance letter

**DOI:** 10.1371/journal.pone.0353105.r004

**Authors:** Wanli Zang

PONE-D-25-64152R1

PLOS One

Dear Dr. Han,

I'm pleased to inform you that your manuscript has been deemed suitable for publication in PLOS One. Congratulations! Your manuscript is now being handed over to our production team.

At this stage, our production department will prepare your paper for publication. This includes ensuring the following:

* All references, tables, and figures are properly cited

* All relevant supporting information is included in the manuscript submission,

* There are no issues that prevent the paper from being properly typeset

You will receive further instructions from the production team, including instructions on how to review your proof when it is ready. Please keep in mind that we are working through a large volume of accepted articles, so please give us a few days to review your paper and let you know the next and final steps.

Lastly, if your institution or institutions have a press office, please let them know about your upcoming paper now to help maximize its impact. If they'll be preparing press materials, please inform our press team within the next 48 hours. Your manuscript will remain under strict press embargo until 2 pm Eastern Time on the date of publication. For more information, please contact onepress@plos.org.

You will receive an invoice from PLOS for your publication fee after your manuscript has reached the completed accept phase. If you receive an email requesting payment before acceptance or for any other service, this may be a phishing scheme. Learn how to identify phishing emails and protect your accounts at https://explore.plos.org/phishing.

If we can help with anything else, please email us at customercare@plos.org.

Thank you for submitting your work to PLOS ONE and supporting open access.

Kind regards,

PLOS ONE Editorial Office Staff

on behalf of

Dr. Wanli Zang

Guest Editor

PLOS One
